# Properties of Fiber-Reinforced Geopolymer Mortar Using Coal Gangue and Aeolian Sand

**DOI:** 10.3390/ma17133225

**Published:** 2024-07-01

**Authors:** Yupeng Li, Akelamjiang Maimait, Jianjun Cheng, Yanfu Duan, Yuanqing Chen, Hongguang Dong

**Affiliations:** 1 College of Water Conservancy & Architectural Engineering, Shihezi University, Shihezi 832003, China; l1774786383@163.com (Y.L.); akelamjiang@126.com (A.M.); lttdyf@163.com (Y.D.); chenyq202302@163.com (Y.C.); dhg19990114@163.com (H.D.); 2 Xinjiang Kusha Highway Development Limited Liability Company, Aksu 843000, China

**Keywords:** geopolymer, polypropylene fiber, mechanical properties, flexural toughness, microscopic analysis

## Abstract

Geopolymers, as a novel cementitious material, exhibit typical brittle failure characteristics under stress. To mitigate this brittleness, fibers can be incorporated to enhance toughness. This study investigates the effects of varying polypropylene fiber (PPF) content and fiber length on the flowability, mechanical properties, and flexural toughness of coal gangue-based geopolymers. Microstructural changes and porosity variations within the Fiber-Reinforced Geopolymer Mortar(GMPF) matrix were observed using scanning electron microscope (SEM) and Low field NMR(LF-NMR) to elucidate the toughening mechanism of PPF-reinforced geopolymers. The introduction of fibers into the geopolymer matrix demonstrated an initial bridging effect in the viscous geopolymer slurry, with a 3.0 vol% fiber content reducing fluidity by 5.6%. Early mechanical properties of GMPF were enhanced with fiber addition; at 1.5 vol% fiber content and 15 mm length, the 3-day flexural and compressive strengths increased by 30.81% and 17.4%, respectively. Furthermore, polypropylene fibers significantly improved the matrix’s flexural toughness, which showed an increasing trend with higher fiber content. At a 3.0 vol% fiber content, the flexural toughness index increased by 198.35%. The data indicated that a fiber length of 12 mm yielded the best toughening effect, with an 84.03% increase in the flexural toughness index. SEM observations revealed a strong interfacial bond between fibers and the matrix, with noticeable damage on the fiber surface due to frictional forces, and fiber pull-out being the predominant failure mode. Porosity testing results indicated that fiber incorporation substantially improved the internal pore structure of the matrix, reducing the median pore diameter of mesopores and converting mesopores to micropores. Additionally, the number of harmless and less harmful pores increased by 23.01%, while the number of more harmful pores decreased by 30.43%.

## 1. Introduction

With the accelerated pace of urbanization, the production of ordinary Portland cement (OPC) has reached 4 billion metric tons and is expected to further increase in the next decade. However, traditional cement production not only consumes vast amounts of raw materials but also releases significant amounts of carbon dioxide during the calcination of limestone, causing long-term adverse environmental impacts. To address this challenge, researchers have explored the use of alkali-activated industrial solid wastes, including fly ash (FA), silica fume (SF), ground granulated blast furnace slag (GGBS), coal gangue (CG), and silica fume, to produce geopolymer concrete. Although geopolymer concrete also exhibits issues such as high brittleness and low tensile strength, it offers several advantages over OPC concrete, including a wider range of material sources, lower carbon emissions, higher early strength, and excellent durability.

The mechanical properties of geopolymers are very similar to those of cement, exhibiting excellent compressive strength and relatively poor tensile strength, which results in a certain degree of brittleness. To improve this brittleness, small fibers are typically randomly mixed into traditional concrete to form what is known as fiber-reinforced concrete (FRC). In common brittle materials, once cracks initiate, they tend to propagate rapidly, leading to a sharp decline in the load-bearing capacity of the matrix. However, in FRC, the fibers distributed within the concrete matrix can intercept crack propagation, slowing down or even stopping crack formation. This phenomenon, known as the crack bridging effect, significantly enhances the toughness of the concrete and maintains its load-bearing capacity even after the initial crack appears. Inspired by this approach, researchers have attempted to incorporate fibers into geopolymers to improve their brittleness, achieving varying degrees of success. Table 1 summarizes recent studies on fiber-reinforced geopolymers, providing specific details on their findings.

Intarabut et al. [8] investigated the use of basalt, steel, and polypropylene fibers to enhance fly ash-based geopolymers and found that each fiber type exhibited different failure modes. Steel and polypropylene fibers demonstrated complete bond-slip responses due to their higher ductility, whereas basalt fibers, being brittle, failed through fiber rupture without slip during the pull-out response. Piti [9] addressed the high flexibility but low stiffness of polypropylene fibers by hybridizing them with steel fibers. The results showed that hybridization with steel fibers could improve the flexural performance, toughness, and residual strength of polypropylene fiber-reinforced geopolymers to varying degrees. Ömer [10] studied the effects of polypropylene, steel, and polyamide fibers on the mechanical and durability properties of fly ash-based geopolymers. Experimental results indicated that fiber addition enhanced the mechanical properties, with polypropylene and steel fibers significantly increasing the flexural toughness, while polyamide fibers showed a less pronounced effect. Jiratchaya [11] discovered that incorporating bamboo and glass fibers into metakaolin-based geopolymers effectively enhanced flexural strength. Compared to glass fibers, the rough bamboo fibers formed stronger interlocking structures with the matrix, exhibiting superior bonding capability and increasing flexural strength by 1.9 times with 1% bamboo fiber addition. Zhang Peng et al. [12] found that polypropylene fibers were most effective in enhancing the toughness of fly ash-based geopolymers, achieving a flexural strength of 21.1 MPa at 28 days with a 1.2% fiber content. Mohseni [13] reported a 28% increase in flexural strength with the addition of PF to geopolymer samples. Al-Mashhadani et al. [14] examined the reinforcing effects of polypropylene, steel, and polyvinyl alcohol fibers on fly ash-based geopolymers, concluding that fiber addition did not significantly increase the compressive strength of the mortar. Steel and polyvinyl alcohol fibers increased compressive strength by only 3.37% and 4.26%, respectively, but flexural strength showed significant improvements compared to the control mortar. Guo and Pan [15] found that fiber volume fractions exceeding 0.2% negatively impacted flexural strength, recommending an optimal fiber usage rate of 0.2% for fly ash slag-based geopolymers.

In summary, while research on using fibers to enhance the mechanical properties of cement-based materials has yielded substantial results, studies on the modification of new cementitious materials like geopolymers with fibers remain insufficient. Most research focuses on fly ash-based geopolymers, with few studies examining fiber-reinforced coal gangue-based geopolymers. Therefore, this study explores the effects of different fiber contents and lengths on the workability and mechanical properties of polypropylene fiber-reinforced coal gangue-slag-based geopolymers (GMPF). Additionally, SEM and LF-NMR microscopic characterization methods are used to observe the microstructure within the matrix and investigate the mechanisms by which fibers affect flexural toughness. This research aims to elucidate the mechanical and toughness enhancement effects of polypropylene fibers on coal gangue-based geopolymers, providing references for the application of fiber-reinforced geopolymer composites and the recycling of coal gangue.

## 2. Materials and Methods

### 2.1. Raw Materials

(1) Binder Materials and Aggregates

**Figure 1:**
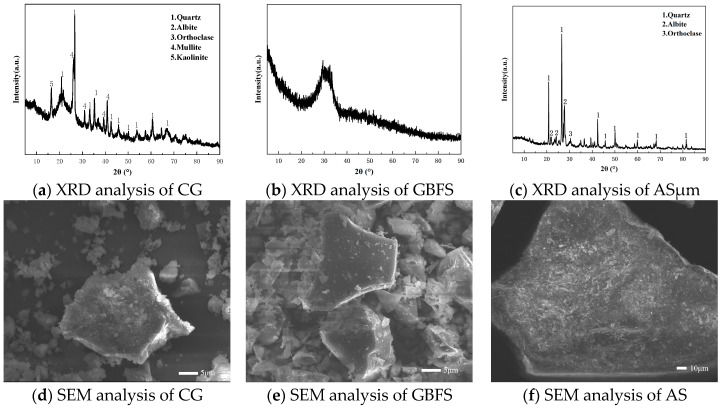
XRD and SEM analyses of raw materials.

**Figure 2:**
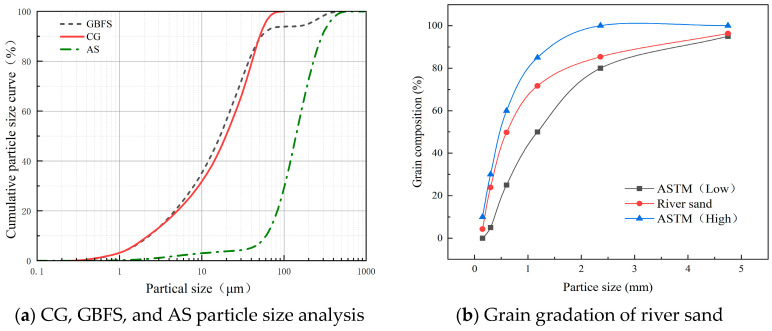
Particle size analysis and particle grading.

The preparation of geopolymers primarily utilizes two raw materials: coal gangue powder (CG) and ground granulated blast furnace slag (GBFS). Coal gangue powder is a mature industrial product obtained by crushing, ball milling, and high-temperature treatment in a calcination furnace of raw coal gangue. The GBFS is sourced from an industrial park in Northern Xinjiang, China, and is gray-white. According to the GB/T 18046-2017 standard [16], this slag falls under the S75 grade, meeting the standards for use in cement, mortar, and concrete. For mortar preparation, a mixture of aeolian sand (AS) and river sand (RS) is used as aggregates. This combination leverages the hardness and particle shape of aeolian sand with the smoothness and particle size distribution of river sand, aiming to achieve better workability and mechanical strength. The physical and chemical properties of the materials are shown below, and the elemental composition of the raw materials is analyzed by X-ray fluorescence (XRF), as presented in Table 2. Figure 1 illustrates the X-ray diffraction (XRD) and scanning electron microscopy (SEM) analyses of the raw materials. XRD analysis reveals that coal gangue and aeolian sand are mainly composed of quartz, sodium feldspar, and mica phases, while the XRD of slag mainly exhibits a broad hump shape, indicative of an amorphous phase. The particle size analysis of aeolian sand and particle gradation of river sand are shown in Figure 2. The fineness modulus of river sand is 2.77, complying with standard ASTM C33-18 specifications [17].

(2) Alkali Activator

The alkaline activator is prepared by mixing liquid sodium silicate and sodium hydroxide. The original liquid sodium silicate has a modulus (Na_2_O/SiO_2_) of 3.28, with a Na_2_O content of ≥8.35% and a SiO_2_ content of ≥26.54%. Sodium hydroxide is of analytical grade, with a purity of ≥98%, and the water used in the experiment is laboratory-grade purified water. To prepare the activator, the NaOH and Na_2_SiO_3_ solutions are thoroughly stirred, then covered and allowed to age at room temperature for 24 h. Once completely cooled, the solution is ready for use.

(3) Polypropylene Fiber

The main physical and mechanical properties of polypropylene fibers are presented in Table 3.

### 2.2. Experiment Design

The mix parameters listed below are original contributions by the author. Prior to conducting this experiment, the baseline mix parameters for geopolymer mortar were determined through preliminary testing. The mix proportions for preparing fiber-reinforced geopolymer matrices are provided in Table 4. To prepare the specimens, coal gangue and slag powders are first mixed with the corresponding masses of aeolian sand and river sand aggregates and stirred for 2 min. Once mixing is complete, the pre-prepared alkaline activator solution and water are added rapidly and stirred for 3–5 min. After observing uniform mixing of the slurry, the geopolymer precursor is obtained. The precursor is then slowly poured into molds with dimensions of 40 mm × 40 mm × 160 mm, compacted, covered, and allowed to cure for 24 h. After demolding, the specimens are placed in a standard curing chamber at a temperature of 20 ± 2 °C and a humidity of 95% until the specified age for testing. The specific experimental procedures are outlined in Table 5.

### 2.3. Testing Methods

#### 2.3.1. Mechanical Performance Testing and Flowability Testing

Flowability testing follows the recommended method outlined in GB/T 2419-2005 “Test methods for fluidity of cement mortar” [18]. Initially, the mortar is poured into the truncated cone mold in two batches. Subsequently, the truncated cone mold is quickly lifted, allowing the mortar to flow freely on the flow table. The flow table is then activated to vibrate 25 times at a frequency of once per second. After the completion of the vibration, the diameters in two perpendicular directions are measured using calipers, and the average diameter is taken as the flowability result for the mortar batch.

After curing for 3, 7, and 28 days, flexural and compressive strength tests are conducted using a hydraulic servo-controlled universal testing machine. Three prism specimens are taken for flexural strength testing, and the average of three test results is recorded. During flexural strength testing, the loading rate is 1 mm/min until contact with the specimen surface is made, then it increases to 40 N/s. For compressive strength testing, the broken halves of the specimens are placed in fixtures, and the loading rate is set at 2.4 kN/s. The final compressive strength result is the average of six test results.

#### 2.3.2. Flexural Toughness Testing

Flexural toughness is evaluated using the Japanese Society of Civil Engineers (JSCE) SF4 assessment method. The flexural toughness and flexural toughness coefficient are calculated according to the following methods.

The method for calculating flexural toughness is as follows:

(1) $$T_{b}=\int p(\delta )d_{\delta }$$

where $T_{b}$ is the flexural toughness, which is the area under the load–displacement curve at the corresponding deflection. $P\left(\delta \right)$ is the load–displacement curve during the three-point bending test.

The method for calculating the flexural toughness coefficient is as follows:

(2) $$\sigma_{b}=\frac{T_{b}}{\delta_{tb}}\times \frac{L}{bh^{2}}$$

where $\sigma_{b}$ is the flexural toughness coefficient; $T_{b}$ is the flexural toughness; $\delta_{tb}$ is the deflection at 1/150 L; and L, b, and h are the length, width, and height of the specimen, respectively.

#### 2.3.3. Scanning Electron Microscopy (SEM) and Low-Field Nuclear Magnetic Resonance (LF-NMR) Analysis

Samples, after 28 days, are soaked in anhydrous ethanol for 24 h to halt hydration reactions. Subsequently, the samples are dried to a constant weight. The central portion of the remaining samples is then crushed into fragments approximately 5 mm in size for scanning electron microscopy (SEM) analysis. Half of the fractured specimens are used for volume measurement and then subjected to low-field nuclear magnetic resonance (LF-NMR) analysis. The internal microstructure of the samples is observed using a Japanese JSM IT-800 field emission scanning electron microscope (JEOL, Tokyo, Japan) under an acceleration voltage of 15 kV. The China NIUMAG MacroMR large-aperture nuclear magnetic resonance imaging analysis system (MacroMR02-110H-D, Suzhzhou Niumag Analytical Instruments Corporation, Suzhou, China) is utilized to analyze the internal porosity, pore size distribution, and pore throat distribution of the samples. The magnetic field strength is set to 0.3 T, and the frequency field is set to 23 MHz.

## 3. Results

### 3.1. Flowability

**Figure 3:**
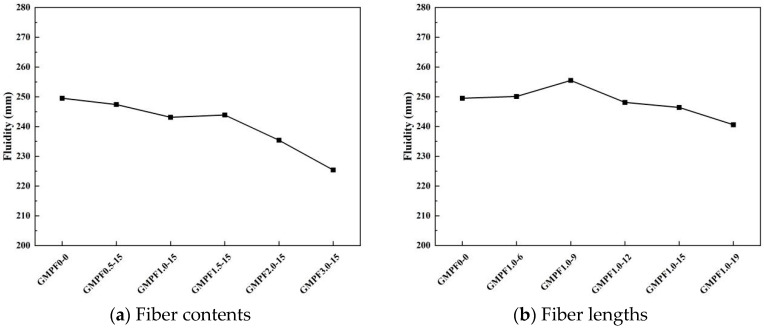
Influence of different fiber contents and fiber lengths on flowability.

The impact of different fiber dosages on flow performance is illustrated in Figure 3a. The dosage of 15 mm polypropylene fibers increases from 0 to 3 vol%, leading to a gradual decrease in flow performance. When the dosage reaches 3 vol%, the flow performance is at its lowest, with a decrease of 9.6% compared to the unfibered state. It can be observed from the graph that within the range of 0–1.5 vol% fiber dosage, the decrease in flowability is relatively moderate, ranging from 0.85% to 2.56%, compared to the reference group. However, when the fiber dosage exceeds 1.5 vol%, the decrease in flow performance accelerates noticeably, with flowability decreasing by 2.24% and 5.6% for the GMPF2.0-15 and GMPF3.0-15 groups, respectively, compared to the reference group. Figure 3b illustrates the effect of different fiber lengths on flow performance with a fiber dosage of 1 vol%. From the GMPF0-0 group to the GMPF1.0-9 group, there is a slight improvement in flow performance. When the fiber length is 9 mm, the flowability of the GMPF slurry increases by 2.4% compared to the unfibered state. However, with fiber lengths of 12 mm, 15 mm, and 19 mm, flowability gradually decreases. The GMPF1.0-19 group exhibits the lowest flowability, with a decrease of 5.8% compared to the GMPF1.0-9 group. Overall, the effect of different fiber lengths on flow performance is relatively minor when the fiber dosage is less than 1.5 vol%. This is mainly due to the hydrophobic nature of polypropylene fibers, which typically exhibit low water absorption and are not easily wetted by water. Consequently, water droplets form on the fiber surface and quickly slide off, providing lubrication in the system [19]. Additionally, short fibers in the matrix can fully encapsulate the remaining water to wrap the aggregates, thereby enhancing flow performance. However, for long fibers, more polymer matrix adheres to the surface, demonstrating a preliminary bridging effect. This effect is more significant for the inherently viscous geopolymer precursor, leading to a 3.8% decrease in flow performance compared to the reference group for the GMPF1.0-19 group.

### 3.2. Mechanical Performance

#### 3.2.1. Influence of Different Fiber Contents on Mechanical Strength

**Figure 4:**
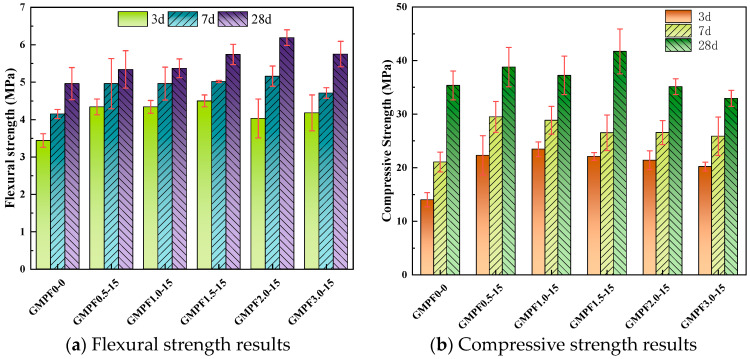
Mechanical strength results with different fiber contents.

Figure 4a presents the flexural strength results at 3d, 7d, and 28d for different fiber dosages. It can be observed that the inclusion of polypropylene fibers leads to a significant improvement in the early flexural strength of the GMPF specimens. The GMPF1.5-15 group achieves a flexural strength of 4.55 MPa at 3d, representing a 30.81% increase compared to the GMPF0-0 group. The GMPF2.0-15 group exhibits the highest flexural strength at 7d and 28d, reaching 5.1 MPa and 6.1 MPa, respectively, corresponding to increases of 24.33% and 11.53% compared to the reference group. As the curing period increases, the efficiency of polypropylene fibers in enhancing the flexural strength of geopolymer matrix gradually diminishes. Comparisons among different fiber dosage groups reveal a trend of initially increasing and then decreasing flexural strength with increasing fiber dosage. The highest flexural strength is achieved at a fiber dosage of 2.0 vol%, with a 25.65% increase compared to the reference group. When the fiber dosage is between 0.5 vol% and 1.0 vol%, the improvement in flexural strength at 28d is extremely limited. Additionally, the flexural strength of the GMPF samples with a 3.0 vol% polypropylene fiber dosage shows a decreasing trend, with a 7.1% decrease compared to the preceding group. Xu et al. [20] observed a similar phenomenon with basalt fiber in fly ash-based geopolymer, where an excessive dosage of basalt fiber adversely affected the flexural strength.

Figure 4b demonstrates the effect of different fiber dosages on compressive strength. At 3d and 7d, the inclusion of fibers positively contributes to the growth of compressive strength. The results indicate that at 3d, the GMPF0.5-15 sample achieves the highest compressive strength of 23.46 MPa, compared to 14.01 MPa for the control sample. At 28d, the sample with a 1.5 vol% polypropylene dosage exhibits the highest compressive strength, with a 17.4% increase compared to the control group. The data suggest that when the polypropylene fiber dosage exceeds 1.5 vol%, further increases result in a decreasing trend in compressive strength. Shaikh et al. [21] found a decrease in compressive strength with PE fibers in geopolymer; however, unlike the substantial decrease in strength observed with PE fibers, this study indicates that PP fibers do not cause a cliff-like drop in matrix compressive strength. It is well known that polypropylene fibers are primarily randomly distributed within the matrix. When the matrix is subjected to tensile stress, the high modulus of elasticity of the fibers enables them to both disperse stress and bear some of the load, thereby enhancing the flexural and compressive strength of the sample. On the other hand, increasing the fiber dosage introduces more bonding interfaces and micro-pores, which affect the polymerization reaction of the geopolymer. Additionally, excessive fiber incorporation will disrupt the uniform distribution of fibers within the matrix, adversely affecting the mechanical strength of the matrix.

#### 3.2.2. Influence of Different Fiber Lengths on Mechanical Performance

**Figure 5:**
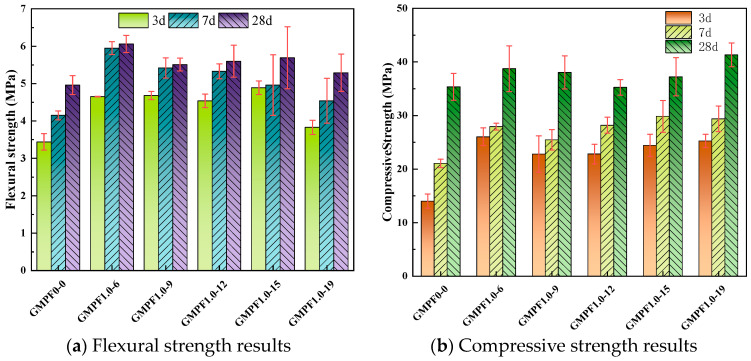
Mechanical strength results with different fiber lengths.

Figure 5a illustrates the flexural strength results for different fiber lengths. Similar to the previous section, the enhancement of early flexural strength by 1 vol% polypropylene fibers is remarkable. The GMPF1.0-6 group achieves the highest flexural strength at 28d, reaching 6.06 MPa, representing a 22.17% increase compared to the reference group. Furthermore, the data indicate that longer fibers do not necessarily result in higher flexural strength. When the fiber length ranges from 9 mm to 19 mm, the flexural strength fluctuates around 5.5 MPa, showing an 11.1% increase compared to the reference group.

Figure 5b depicts the influence of fiber length on compressive strength. The results indicate that fibers of various lengths positively affect compressive strength at 3d and 7d. At 7d, the compressive strength with 15 mm polypropylene fibers is 41.8% higher than that without fibers. The enhancement in flexural strength diminishes with increasing curing time, with the difference in compressive strength between fiber-reinforced samples and the reference group being relatively small at 28d, with a 5.31% increase observed in the GMPF1.0-15 group. This characteristic is similar to the conclusion drawn for polypropylene fibers in cementitious materials, where the influence of fiber length on compressive strength is weak [22]. The experimental results suggest that 6 mm long polypropylene fibers are more suitable for coal gangue-based geopolymer. It was found during mixing that short fibers disperse more uniformly in the geopolymer slurry and can form a better interlocking structure with the geopolymer matrix, exhibiting stronger mechanical properties. Additionally, polypropylene fibers can partially inhibit crack formation in the matrix under compression, thereby enhancing compressive strength. However, due to their lower modulus of elasticity, they cannot prevent cracks from continuing to propagate once they appear, resulting in a weaker enhancement in compressive strength.

### 3.3. Flexural Toughness

#### 3.3.1. Load–Deflection Curve

**Figure 6:**
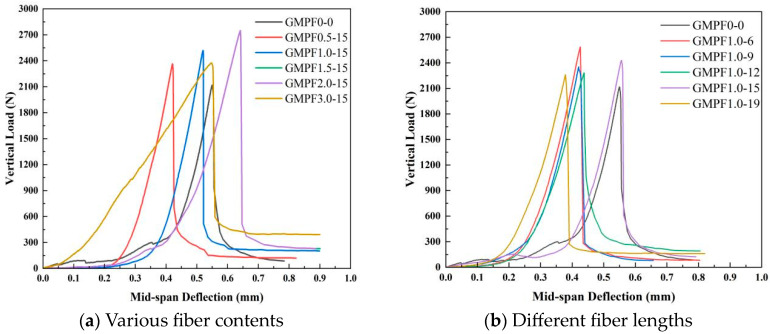
Load-deflection curves of GMPF specimens under bending.

The bending load–deflection curves for specimens with different fiber contents are presented in Figure 6a. The data indicate a gradual increase in both maximum bending force and maximum deflection with increasing fiber content. It is observed that at a polypropylene fiber content of 2.0 vol%, the maximum bending force increases by 29.8%, and the maximum deflection increases by 36.47%. Additionally, particular attention should be paid to the change in curve morphology at a polypropylene fiber content of 3.0 vol%. In the GMPF3.0-15 group, prior to reaching the maximum bending force, the load–deflection curve is nearly linear, with a smaller slope, and the peak at the maximum bending force is smoother, indicating that the specimen absorbs significantly more energy before failure compared to the other groups, highlighting the most pronounced improvement effect of fibers on the matrix.

The bending load–deflection curves for different fiber lengths are illustrated in Figure 6b. The change trends in the curves are relatively consistent as the fiber length increases from 6 mm to 19 mm. The load–deflection curve can be roughly divided into three stages: initially, when the specimen begins to bend, the testing rate is slow, and the curve exhibits the “cushioning zone”, with relatively low stress but considerable displacement; subsequently, the bending force rapidly increases while the displacement changes only slightly, indicating the elastic stage of bending; finally, when reaching the maximum bending force, cracks appear, the bending force drops rapidly, and the displacement continues to increase, entering the strain-softening stage, leading to specimen failure. The experimental results demonstrate that even with an increase in fiber content from 0 to 3 vol% and fiber length from 6 mm to 19 mm, the GMPF composite material does not exhibit strain-hardening characteristics similar to fiber cementitious materials; its load–deflection curve displays typical brittle failure characteristics. Studies by Lin et al. [23]. suggest that compared to steel and polyoxymethylene fibers, polypropylene fibers have poorer bonding ability with the matrix and limited fiber bridging capacity, which may explain the insignificant change in the failure mode.

In summary, the incorporation of polypropylene fibers into coal gangue-based geopolymer composite materials has a beneficial effect on flexural performance. The fibers prevent crack propagation within the geopolymer mortar phase through bridging effects, resist stress applied to the specimens, and transmit stress to regions of the mortar with stronger resistance to bending [24]. Furthermore, the composite material absorbs more energy when subjected to stress. Once cracks appear in the specimen, the fibers fracture and debond from the matrix one by one, dissipating energy in the process, effectively enhancing the material’s flexural strength.

#### 3.3.2. Influence of Fibers on Flexural Toughness Coefficient

The flexural toughness was assessed using the Japanese Society of Civil Engineers (JSCE) SF4 evaluation method, as described in Section 2.3.2. Toughness can be regarded as the energy absorbed by the specimen when a certain rotation occurs and can be represented by the area under the load–deflection curve. Residual strength refers to the strength of the specimen when the load decreases to a stable value after surpassing its peak in the load–deflection curve. The calculated results for the relevant parameters are presented in Table 6.

**Figure 7:**
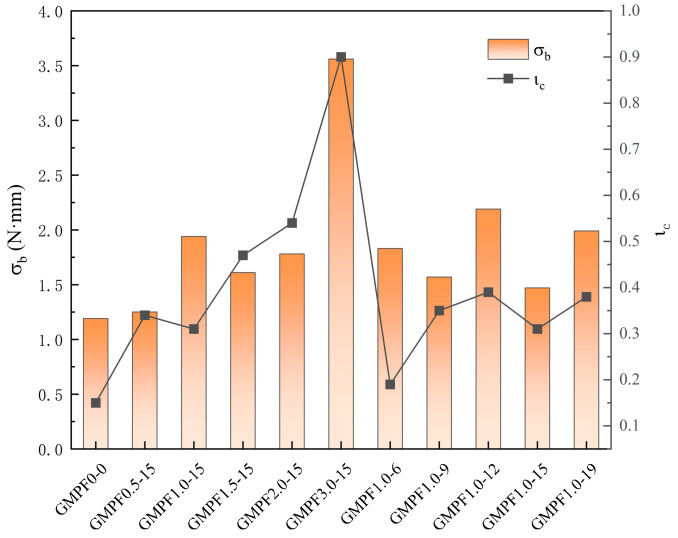
Flexural toughness coefficient and residual strength of GMPF.

The results of the GMPF toughness coefficient calculations are presented in Figure 7, illustrating a noticeable enhancement in the flexural toughness coefficient with fiber incorporation. When the fiber content is 1.0 vol%, the flexural toughness coefficient increases by 63.02% compared to the control group. Similarly, at a fiber content of 3.0 vol%, the flexural toughness coefficient experiences a remarkable increase of 198.35%. This indicates a significant reinforcement effect of polypropylene fibers on the flexural toughness of geopolymer mortar, with the enhancement effect increasing proportionally with the fiber content, mirroring findings similar to those reported by Sukontasukkul et al. [9] in fly ash-based geopolymers. The effect of fiber length on toughness coefficient variations exhibits a fluctuating pattern, with enhancements ranging from 23.52% to 84.03% compared to the GMPF0-0 group as the length increases from 6 mm to 19 mm. The enhancement effect of polypropylene fiber length on flexural toughness is notably significant compared to the control group, while variations within the same group are minor. For instance, compared to the GMPF1.0-12 group, the GMPF1.0-15 group experiences a 48.97% increase in flexural toughness coefficient. Experimental results demonstrate that a fiber length of 12 mm yields the most significant enhancement in flexural toughness.

Figure 6 illustrates the relationship between residual strength and different fiber contents and lengths. Experimental results indicate an increase in residual strength with higher fiber content. Compared to the control group, residual strength increases by 123.24%, 103.05%, 206.72%, 249.54%, and 484.09% with fiber contents of 0.5 vol%, 1.0 vol%, 1.5 vol%, 2.0 vol%, and 3.0 vol%, respectively. Simultaneously, the influence of fiber length on residual strength grows with increasing length. As fiber length increases from 6 mm to 19 mm, the rate of residual strength improvement ranges from 103.05% to 152.59%. The effect of fiber length on the growth of flexural residual strength appears relatively gradual, attributed to the enhancement of post-peak performance by longer fibers. Longer fibers can provide more interlocking force and energy absorption after the material reaches its peak strength, thereby strengthening the interface bonding between fibers and the matrix, consequently delaying material failure.

In summary, residual strength denotes a specimen’s capacity to resist loads after failure, and the inclusion of fibers significantly improves the post-failure stress condition of fly ash-based geopolymers, allowing for a residual flexural strength even after failure. Moreover, considering that fibers in mortar form a robust supporting structure with aggregates, this enhances the overall integrity of the matrix structure, reduces significant deformation of mortar, and mitigates the brittle failure characteristics of fly ash-based geopolymers.

### 3.4. Microscopic Analysis

#### 3.4.1. SEM Analysis

**Figure 8:**
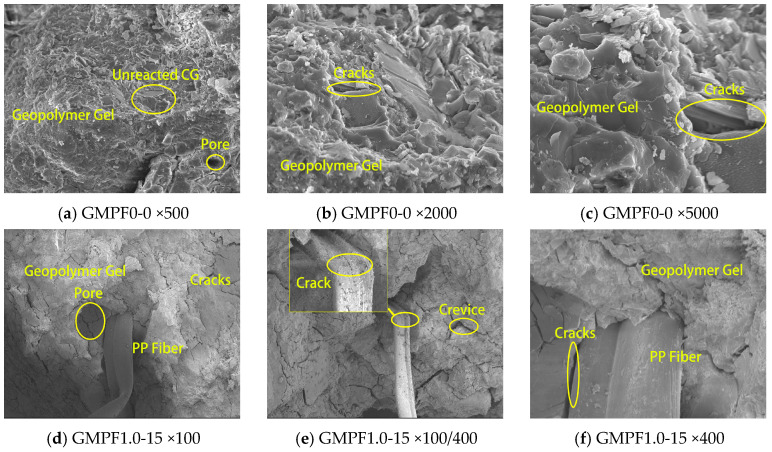
SEM images of GMPF at different magnifications.

SEM analysis was conducted on the non-fiber-incorporated group, GMPF0-0, and the fiber-incorporated group, GMPF1.0-15, with the results depicted in Figure 8. From the SEM images of the GMPF0-0 group, it is evident that the compressed GMPF matrix is surrounded by a large amount of amorphous polymer cementitious material. The gel is predominantly irregularly clustered, covering the surface of the matrix, thus densifying the internal structure of the matrix. Additionally, in Figure 8a, unreacted CG particles are observed, existing within the matrix as fillers, reducing micro-defects such as cracks and voids, consequently enhancing the compactness of the structure. Nevertheless, Figure 8b and Figure 8c still reveal the presence of numerous cracks, where larger and wider cracks are primarily due to compressive failure of the matrix, while finer cracks result from significant curing shrinkage of the GMPF matrix itself.

Figure 8d–f illustrate the microscopic structure of GMPF fibers with a content of 1.0 vol% and a length of 15 mm. It can be observed that the addition of fibers significantly reduces the occurrence of micro-cracks. The highly elastic PP fibers are distributed within the matrix, optimizing the stress distribution within the matrix, mitigating stress concentration at matrix defects, thus enhancing the compressive strength of the specimens. This is consistent with the trend observed in macroscopic mechanical property changes; when the fiber content is 1.0 vol%, compressive strength increases by 5.31%, and flexural strength increases by 8.2%. However, due to the presence of non-polar chemical bonds on the surface of PP fibers, they exhibit an extremely hydrophobic surface structure. This hydrophobic surface weakens the interaction between PP fibers and the matrix, resulting in the existence of partial cracks and voids between the fiber–matrix interface, leading to a decrease in the density of the matrix structure.

In Figure 8d,f, it can be observed that there is no adherent polymer gel on the fiber surface, and numerous small cracks and voids exist between the fiber and the matrix, weakening the interface bonding ability of the fiber and the matrix, thus diminishing the bridging effect of the fiber. In Figure 8e, it is observed that the connection ends of the fibers exhibit significant tearing and friction marks after loading, with multiple longitudinal cracks appearing on the fiber surface, indicating that polypropylene fibers still possess strong mechanical interlocking capability with the geopolymer matrix. This may be a reason for the slight increase in flexural strength of GMPF specimens but a significant improvement in flexural toughness.

#### 3.4.2. Pore Size and Pore Throat Distribution

The pore size and pore throat distribution of the GMPF specimens were analyzed using the low-field nuclear magnetic resonance test method, and the analysis results are presented below.

**Figure 9:**
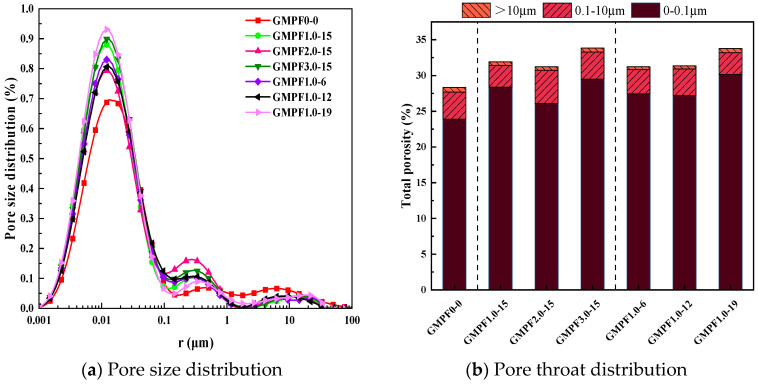
Distribution of different GMPF pore types and pore throat sizes.

Figure 9a illustrates the variation in pore distribution among different groups of GMPF. It can be observed from the figure that the pore distribution curve roughly consists of three peaks, with peak values corresponding to pore sizes of 0.01 μm, 0.5 μm, and 10 μm, respectively. Therefore, following the classification method for pore sizes in cementitious materials, pores ranging from 0 to 0.1 μm are termed micropores, pores ranging from 0.1 to 10 μm are termed mesopores, and pores larger than 10 μm are termed macropores [25]. Additionally, it is noted that peak pores are mainly distributed around the peak near 0.01, followed by the peak near 0.5 μm, while the distribution of pores near 10 μm is minimal. This indicates that the overall pore structure of the GMPF matrix is predominantly composed of micropores, with relatively fewer mesopores and macropores, suggesting a compact overall structure. Compared to the reference group, with the increase in fiber content, the content of micropores in the matrix gradually increases, and the median pore size peak of mesopores shifts to the left, while the median pore size peak corresponding to macropores shows a trend of rightward movement. This indicates that fibers in the matrix can effectively fill the pores of mesopores, converting mesopores with pore sizes ranging from 0 to 10 μm into mesopores with smaller pore sizes ranging from 0.1 to 1 μm and micropores below 0.1 μm. The trend of an expanding pore size of macropores is due to the segmented mesopores merging with other pores in the geopolymer precursor state, transforming mesopores into macropores. The impact of different fiber lengths on pore structure has not varied significantly, with a more significant effect observed on micropores. Generally, longer fibers lead to a higher content of micropores, while changes in mesopores and macropores are minimal, indicating that the internal pore structure of the matrix has not deteriorated.

Table 7 presents the porosity and distribution of harmful pores in GMPF specimens. The definition of harmful pores follows the classification method summarized by Academician Wu Zhongwei, categorized based on the pore size’s damaging effect on the mechanical strength of concrete structures, into harmless pores (0 ≤ r < 20 nm), slightly harmful pores (20 ≤ r < 100 nm), harmful pores (100 ≤ r < 200 nm), and multiple harmful holes (r ≥ 200 nm). The data results show that the porosity increases with the increase in fiber content. When the fiber content increases to 1.0 vol%, 2.0 vol%, and 3.0 vol%, the total porosity of the matrix increases by 11.89%, 9.34%, and 18.64%, respectively. This is because the addition of fibers during specimen preparation introduces some small air bubbles, which are difficult to compact, thus remaining in the matrix. However, considering the combination with mechanical properties, the increase in porosity has not excessively reduced the compressive strength of the GMPF matrix. Its compressive strength still shows improvement compared to the reference group, indicating that the enhancement effect of PP fibers on the mechanical properties of the GMPF matrix outweighs the weakening effect caused by the increase in porosity. On the other hand, due to the hydrophobic nature of polypropylene fiber surfaces, there are gaps between the fibers and the matrix bonding surface, which form pores during geopolymer curing. This phenomenon has been observed in the SEM images of fiber–matrix bonding surfaces in Section 3.4.2. The analysis shows that the longer the fibers, the more pronounced this phenomenon, and more bubbles are introduced when fibers are added. Therefore, when the fiber length is 19 mm, the porosity increases by 18.6% compared to the reference group.

The calculation of the harmfulness of GMPF pore sizes reveals that pores in the geopolymer matrix primarily appear in the form of harmless and slightly harmful pores, with harmful and multiple harmful holes accounting for only 4.67%. This indicates that the internal structure of the fly ash-based geopolymer matrix is sound, and the polymerization reaction is sufficient, allowing the formed geopolymer gel to effectively fill harmful pores, forming a robust skeletal structure. With the addition of polypropylene fibers, the pore structure of the fiber–geopolymer composite material is significantly improved. When the fiber content increases from 1.0 vol% to 3.0 vol%, the proportion of harmless and slightly harmful pores increases from 23.94% to 28.37%, 26.09%, and 29.45%, respectively, while the proportion of multiple harmful holes decreases from 4.14% to 2.88%, 3.86%, and 3.42%. This is mainly attributed to the filling effect of the fibers in the matrix, converting multiple harmful holes into less harmful, slightly harmful, and harmless pores, optimizing the internal pore structure of the specimens, and increasing the density of the GMPF matrix. Therefore, the fiber-incorporated group exhibits higher mechanical performance. When the fiber content is fixed at 1.0 vol%, varying fiber lengths show a more significant effect on pore structure changes. When the fiber lengths are 6 mm, 12 mm, 15 mm, and 19 mm, the proportion of harmless and slightly harmful pores increases from the original 23.94% to 27.45%, 27.2%, 28.37%, and 31.43%, respectively, while the proportion of multiple harmful holes decreases from 4.14% to 2.99%, 3.22%, 2.88%, and 1.72%, respectively. It is evident that longer fiber lengths result in more harmless and slightly harmful pores and fewer multiple harmful holes, indicating a better internal pore structure of the matrix. Overall, the optimal pore structure is achieved when the polypropylene fiber content is 1 vol% and the length is 19 mm, resulting in the best mechanical strength, consistent with the conclusions in Section 3.2.2.

## 4. Conclusions

(1) As the PP fiber content increased, more geopolymer paste adhered to the fiber surface, leading to a more pronounced bridging effect in the viscous geopolymer precursor and reducing the fluidity of the GMPF slurry. When the fiber content was less than 1.5 vol%, the fiber length did not significantly affect the flow performance.
(2) The 6 mm polypropylene fibers are more suitable for geopolymers as they disperse more uniformly, forming better interlocking structures and enhancing mechanical properties. The fibers significantly improve the post-failure stress performance and flexural strength of the material, creating a robust support structure within the mortar. This reduces deformation and mitigates brittle failure.
(3) Tears and friction marks were observed at the ends of the fibers where they connect with the matrix after loading, indicating that the fibers effectively suppressed the formation of microcracks. This optimized the internal stress distribution, reducing stress concentration at defects and significantly enhancing the flexural toughness.
(4) LF-NMR results indicated that the incorporation of polypropylene fibers improved the internal pore structure of the geopolymer. As the fiber content increased, the number of small pores increased, and the median pore size of medium and large pores expanded. While fiber length did not alter the overall pore structure, it significantly affected the micropores.

## Figures and Tables

**Table 1:** Research progress of fiber geopolymers in recent years.

Study	Fiber Type	Fiber Content	Fiber Length	Geopolymer Matrix	Key Findings
Li et al. [1]	Steel fiber	0.5 vol%	13 mm	GBFS-based	Steel fibers are less susceptible to corrosion, while effectively enhancing both compressive and flexural strength.
Zhang et al. [2]	Carbon fiber	0.6 vol%	6 mm	Fireclay-based	Carbon fibers with a length of 6 mm demonstrated better crack initiation toughness and resistance to unstable cracking.
Zhang et al. [3]	Polypropylene fiber	1.0 vol%	35 mm	FA- and GBFS-based	Polypropylene (PP) fibers exacerbated the adverse effects of frost heave stress and thermal stress on the geopolymer matrix (GPM), leading to a reduction in frost resistance.
Li et al. [4]	Polyethylene fiber	1.5 vol%	12 mm	FA-, SF-, and GBFS-based	High fiber content of polyethylene fiber (PEF) significantly enhanced the compressive strength and fracture toughness of the specimens.
Zhang et al. [5]	Polypropylene fiber and polyethylene fiber	2.0 vol%	12 mm	FA- and GBFS-based	The addition of PP fibers reduced the tensile strength and crack control ability. However, with a PP fiber replacement rate of 50%, the highest tensile strain capacity was achieved (9.66%).
Zhao et al. [6]	Sisal fiber	1.0 vol%	20 mm	GBFS-based	The addition of SF reduced the density and electrical conductivity (EC), while simultaneously increasing the porosity, long-term flexural strength, and compressive strength.
Liu et al. [7]	Glass fiber	0.6 vol%	9 mm	FA-based	Glass fibers significantly improved the splitting tensile strength and enhanced the pore characteristics, contributing to a more uniform pore distribution.

**Table 2:** Chemical composition analysis of raw materials.

Materials	SiO_2_	Al_2_O_3_	CaO	Fe_2_O_3_	Mg_2_O_3_	Na_2_O	K_2_O	Other
CG	53.45	43.46	0.317	0.781	0.11	0.17	0.291	1.421
GBFS	32.85	8.62	45.16	0.63	5.69	1.08	0.497	5.473
AS	59.3	11	8.42	9.125	1.52	3.76	3.45	3.425

**Table 3:** Principal physical and mechanical properties of polypropylene fibers.

Equivalent Diameter (μm)	Tensile Strength (MPa)	Initial Modulus (MPa)	Elongation at Break (%)	Density (g/cm^3^)
38	550	4.7 × 10^3^	85	0.91

**Table 4:** Base mix ratio of geopolymer preparation (g).

Coal Gangue	GBFS	Water	Na_2_SiO_3_	NaOH	Aeolian Sand	River Sand
270.00	270.00	142.83	198.09	37.09	540.00	540.00

**Table 5:** Experimental protocol for fiber-reinforced geopolymer.

Specimen No.	Coal Gangue (g)	GBFS(g)	Water(g)	Alkali Activator (g)	Fiber Content (g)	Fiber Length (mm)
GMPF0-0	270.00	270.00	142.83	235.18	0	0
GMPF0.5-15	270.00	270.00	142.83	235.18	1.17	15
GMPF1.0-15	270.00	270.00	142.83	235.18	2.33	15
GMPF1.5-15	270.00	270.00	142.83	235.18	3.50	15
GMPF2.0-15	270.00	270.00	142.83	235.18	4.66	15
GMPF3.0-15	270.00	270.00	142.83	235.18	6.99	15
GMPF1.0-6	270.00	270.00	142.83	235.18	2.33	6
GMPF1.0-9	270.00	270.00	142.83	235.18	2.33	9
GMPF1.0-12	270.00	270.00	142.83	235.18	2.33	12
GMPF1.0-15	270.00	270.00	142.83	235.18	2.33	15
GMPF1.0-19	270.00	270.00	142.83	235.18	2.33	19

**Table 6:** Results of toughness and related parameters calculation.

Specimen No.	N_cu_ (N)	Δ_cu_ (mm)	τ_cu_ (MPa)	T_b_ (N·mm)	$\sigma_{b}$ (N·mm^−2^)	τ_c_ (MPa)
GMPF0-0	2117.6	0.55	4.96	262.095	1.19	0.15
GMPF0.5-15	2259.2	0.619	5.30	308.739	1.25	0.34
GMPF1.0-15	2365.4	0.421	5.54	326.805	1.94	0.31
GMPF1.5-15	2518.8	0.52	5.90	334.398	1.61	0.47
GMPF2.0-15	2748.8	0.641	6.44	456.190	1.78	0.54
GMPF3.0-15	2374.4	0.547	5.57	779.261	3.56	0.90
GMPF1.0-6	2583.6	0.426	6.06	312.085	1.83	0.19
GMPF1.0-9	2352.2	0.421	5.51	264.699	1.57	0.35
GMPF1.0-12	2281.2	0.437	5.35	383.111	2.19	0.39
GMPF1.0-15	2427.2	0.556	5.69	326.805	1.47	0.31
GMPF1.0-19	2257.8	0.378	5.29	300.754	1.99	0.38

Note: N_cu_ represents the maximum bending force (N); Δ_cu_ denotes the corresponding deflection at the maximum bending force (mm); τ_cu_ stands for flexural strength (MPa); T_b_ represents the absolute toughness value (N·mm); $\sigma_{b}$ denotes the flexural toughness coefficient (N·mm^−2^); and τ_c_ signifies residual strength (MPa).

**Table 7:** Porosity and distribution of harmful pores in GMPF.

Specimen No.	Total Porosity (%)	Harmless Hole (%)	Slightly Harmful Pores (%)	Harmful Hole (%)	Multiple Harmful Holes (%)
GMPF0-0	28.59	15.43	8.49	0.53	4.14
GMPF1.0-15	31.99	20.51	7.86	0.74	2.88
GMPF2.0-15	31.26	18.37	7.72	1.30	3.86
GMPF3.0-15	33.92	20.55	8.94	1.02	3.42
GMPF1.0-6	31.35	19.18	8.27	0.91	2.99
GMPF1.0-12	31.45	18.49	8.71	1.02	3.22
GMPF1.0-19	33.91	29.80	1.63	0.76	1.72

Note: harmless hole (0 ≤ r < 20 nm), slightly harmful pores (20 ≤ r < 100 nm), harmful hole (100 ≤ r < 200 nm), multiple harmful holes (r ≥ 200 nm).

## Data Availability

The original contributions presented in the study are included in the article, further inquiries can be directed to the corresponding author.
